# OSIPI: A Significant Step Towards Reproducible MR Biomarkers

**DOI:** 10.1002/jmri.29414

**Published:** 2024-04-27

**Authors:** John C. Waterton, James P.B. O'Connor

**Affiliations:** ^1^ Bioxydyn Ltd Manchester UK; ^2^ Division of Informatics Imaging & Data Sciences University of Manchester Manchester UK; ^3^ Division of Radiotherapy and Imaging The Institute of Cancer Research London UK

Clinical research and evidence‐based medicine demand metrics which are objectively defined and comparable across centers and are known by the NIH and FDA as “biomarkers.”^1^ These measurements have putative uses that include but are not limited to: assessing patient prognosis; predicting therapeutic benefit; assessing biological response while on therapy; and monitoring safety in patients receiving interventions.

MRI is extraordinarily rich in such biomarkers, particularly those which are intensity‐based variables, such as relaxation times and measures of perfusion, diffusion, and other dynamic processes. Many thousands of research studies are published every year that report new MRI biomarkers, validate existing MRI biomarkers, or offer evidence for benefit in the clinical adoption of MRI biomarkers.^2^

Sadly, much of this academic innovation has failed to translate to impact personalized healthcare and evidence‐based medicine.^3^ It is true that the MRI community has had some success in determining the repeatability (i.e. same subject and same scanner) of some measurements, enabling clinical trials of investigational therapies, particularly single center. For example, DCE‐MRI K^trans^ can be considered a reliable indicator of biological response in oncology, in that reductions in this biomarker are universally demonstrated in drugs that have an active anti‐vascular mechanism of action.^4^ However, where reproducibility is concerned (i.e. showing that measurements are comparable when acquired on different makes and models of scanner using different pulse sequences and analysis software)—our community has failed to deliver. Lack of multicenter reproducibility has prevented DCE‐MRI K^trans^ from translating into the clinic as a predictive biomarker, since its absolute value depends substantially on the hardware and software used to derive the biomarker at any given research site.

Similar examples abound. In hepatology, the value of liver parenchyma T_1_ has been well demonstrated in numerous studies conducted in the past four decades, particularly for the assessment of fibrosis,^5^ but there remains huge variation between normal liver T_1_ values measured by different investigators: the between‐center (reproducibility) variance is about 3‐fold greater than the between‐subject variance and is a massive 75‐fold greater than the repeatability variance.^6^ Without reproducibility, investigators cannot safely combine MRI measurement data from different centers to create predictive biomarkers for personalized healthcare or to develop evidence‐based medicine. This is why so few MRI biomarkers have achieved the status of companion diagnostics.^7^

In recent years, ISMRM has fostered several initiatives to address this “reproducibility crisis” found within our own community. Notable among these has been the Open Science Initiative for Perfusion Imaging (OSIPI), and recent publications including lexicons for contrast‐agent based perfusion MRI (DCE and DSC‐MRI: CAPLEX) and for arterial spin labeling,^8^, ^9^ mark an important milestone in this initiative.

These two papers establish a lexicon and reporting guidelines, with worked examples, at a much greater level of rigor and detail than previously, which investigators, peer‐reviewers and editors should now use. These should go some way toward revealing, and ultimately preventing, irreproducibility in our methods.

A word of caution, however. It is now over 15 years since the US National Cancer Institute sponsored an open consensus conference at ISMRM in Toronto, leading to authoritative recommendations on the DWI biomarker apparent diffusion coefficient.^10^ This document now has over 2000 citations, but despite containing very clear recommendations that “when performing DW‐MRI for ADC quantification, three or more *b* values should be used. This should include *b* = 0 sec/mm^2^, a *b* value of ≥100 sec/mm^2^, and a higher *b* value of ≥500 sec/mm^2^ (typically 500–750 sec/mm^2^) … [and] …enable calculation of perfusion‐insensitive ADC values by excluding the *b* = 0 sec/mm^2^ image from the ADC calculation,” it is still common for investigators to ignore one or more of these recommendations and continue to add suboptimal data to the literature with ADC values confounded by perfusion.

Similar hazards face the important advances in MRI biomarker quantitation reported in the OSIPI lexicons.^8^, ^9^ These two papers will be widely read and will no doubt be highly cited. But even more importantly the lexicons must be accurately deployed if their aims and objectives are to be fulfilled.
